# Revising quantum optical phenomena in adatoms coupled to graphene nanoantennas

**DOI:** 10.1515/nanoph-2022-0154

**Published:** 2022-06-08

**Authors:** Miriam Kosik, Marvin M. Müller, Karolina Słowik, Garnett Bryant, Andrés Ayuela, Carsten Rockstuhl, Marta Pelc

**Affiliations:** Institute of Physics, Nicolaus Copernicus University in Toruń, Grudziadzka 5, Toruń 87-100, Poland; Institute of Theoretical Solid State Physics, Karlsruhe Institute of Technology (KIT), Karlsruhe 76131, Germany; Joint Quantum Institute, University of Maryland and National Institute of Standards and Technology, College Park 20742, MD, USA; Nanoscale Device Characterization Division, National Institute of Standards and Technology, Gaithersburg 20899, MD, USA; Centro de Física de Materiales, CFM-MPC CSIC-UPV/EHU, Paseo Manuel Lardizabal 5, Donostia-San Sebastián 20018, Spain; Donostia International Physics Center (DIPC), Paseo Manuel Lardizabal 4, Donostia-San Sebastián 20018, Spain; Institute of Nanotechnology, Karlsruhe Institute of Technology (KIT), Karlsruhe 76021, Germany

**Keywords:** adatoms, graphene, nanoantennas, nanoflakes, two-level system

## Abstract

Graphene flakes acting as photonic nanoantennas may sustain strong electromagnetic field localization and enhancement. To exploit the field enhancement, quantum emitters such as atoms or molecules should be positioned in such close proximity to the flake that electron tunneling might influence the optical and electronic properties of the system. However, tunneling is usually not considered if the optical coupling mechanism between quantum emitters and nanoantennas is at focus. This work presents a framework for describing the electron dynamics in hybrid systems consisting of graphene nanoflakes coupled both electronically and optically to adatoms and subject to external illumination. Our framework combines the single-particle tight-binding approach with a nonlinear master equation formalism that captures both optical and electronic interactions. We apply the framework to demonstrate the impact of electron tunneling between the adatom and the flake on emblematic quantum optical phenomena: degradation of coherent Rabi oscillations and quenching of Purcell spontaneous emission enhancement in two-level adatoms in proximity of triangular graphene nanoflakes.

## 1 Introduction

The exceptional optical properties of graphene originate from its unique band structure with linear dispersion represented by the Dirac cones. Small nanoflakes of a few nanometers size sustain resonant response in the optical domain [1] where the flakes can efficiently couple to quantum emitters. However, at these small length-scales the classical description fails and a quantum mechanical approach is required to properly model the optical properties of such systems [2, 3]. To efficiently couple to graphene, quantum emitters should be positioned at distances on the order of nanometers away from the graphene flake. Then, apart from the optical coupling between the flake and the emitter, an additional interaction channel opens up related to the possibility of electron tunneling (or hopping) between them. The effect of electron tunneling was previously confirmed for emitters near metallic nanoantennas in general [4, 5] and near the graphene surface in particular [6–9].

Moreover, graphene offers tunability by electric [10–15] and optical means [16, 17], as well as exploiting various types of doping [18–20]. Doped graphene sustains outstanding plasmonic properties [21–23], i.e., field enhancement accompanied by ultra-small mode volumes [23–25]. This suggests a potential platform to realize tunable atom-thin cavities for quantum optical applications.

In general, photonic nanostructures may tremendously affect the optical properties of quantum emitters. For instance, the most recognized effect is the spontaneous emission enhancement by orders of magnitude called the Purcell effect [26–28]. Another example is the unprecedented light–matter interaction strength whereby energy is alternatively exchanged between the electromagnetic field and the quantum emitter *via* Rabi oscillations at and beyond THz rates [29–32]. The optical coupling between photonic nanostructures and quantum emitters has been widely investigated [33–37], and the same framework has been directly applied to graphene nanoflakes [23]. A mesoscopic framework for the description of plasmon–emitter interactions, which includes electronic spill-out effects via nonlocal corrections to classical models, was described in Refs [38, 39]. However, the influence of the electronic coupling on the optical properties of the coupled system has been so far taken into account by involving relatively demanding and numerically expensive computational tools [4, 40, 41], which involved *ab initio* methods and time-dependent density functional theory techniques.

In this work, we introduce a model that describes the electron dynamics on adatoms located in close proximity to graphene nanoflakes subject to illumination with an external electromagnetic field. The method combines the tight-binding model to characterize eigenstates and eigenenergies of the system with the master equation approach to trace the time evolution of charge density under external illumination, similarly to the method proposed in Ref. [42] and follow-up works [43, 44]. We further include an adatom introducing an additional electron and orbitals to the system, which we have recently used to investigate defects on atomic chains [45].

The framework allows investigating the electro-optical properties of coupled structures made of graphene nanoflakes and adjacent atoms at relatively low numerical cost. In the tight-binding model of graphene flakes, each carbon site introduces one electron free to move across the flake *via* the nearest-neighbor hopping. A rigorous quantum-mechanical description of electron dynamics, in this case, should exploit a many-body approach, where *N*_e_ electrons occupying *N* orbitals require a description in an $\left(\begin{array}{c} N\\ N_{e} \end{array}\right)$ -dimensional Hilbert space. Taking into account the spin degree of freedom, *N* ∼ 2*N*_e_, the scaling of the Hilbert space size with the number of electrons becomes extremely unfavorable. Flakes that are optically active in the visible regime have sizes corresponding to a few hundred atoms, making such straightforward approach too expensive in practice. It is clear that radically simpler methods should be exploited to model the electron dynamics in such large structures. On the other hand, the studied systems are not so large that one could use classical or statistical methods of description, since these require nanoflakes of size scales in the order of 20 nm or larger and fail to properly predict the optical response of smaller structures [2].

Our model is based on the single-particle density-matrix approach in the tight-binding framework [46]. The tight-binding approach provides microscopic insights into the origins of the electronic and optical properties of graphene nanoflakes. Despite its relative simplicity compared to other approaches, such as density functional theory or even more computationally demanding atomistic methods, it captures the physical essence of the material and universal characteristics of adatoms and radicals joined to graphene [47]. Our single-particle approach allows studying electron dynamics and the optical response of nanoflakes containing even hundreds of atoms since the Hilbert space size scales linearly with the number of atoms in the system. However, the favorable scaling comes for a price. In particular, the Coulomb interactions are implemented as nonlinearities through a density-matrix-dependent Hamiltonian. Since the model does not automatically account for the Pauli principle, it has been supplemented with additional constraints which prevent occupations from growing over the allowed threshold. Previously, we have successfully applied the model to investigate the nature of excitations in bare graphene nanoflakes [48, 49] and to adatoms on atomic chains [45].

**Figure 1:**
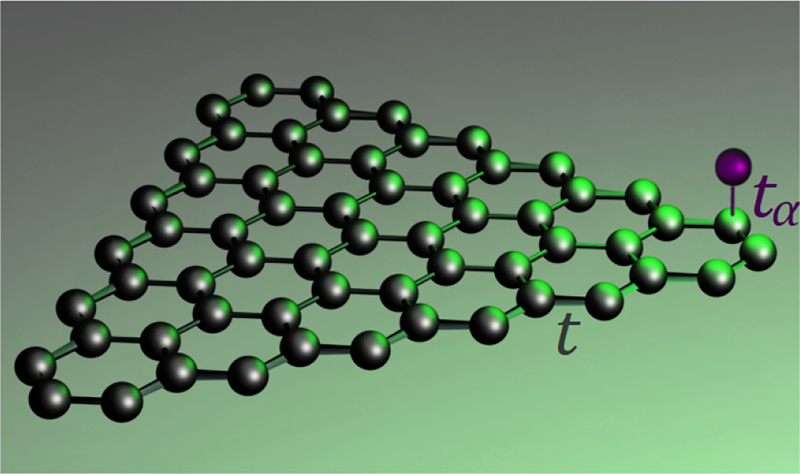
Visualization of the considered hybrid system consisting of a graphene nanoflake and an adatom attached over a particular carbon site.

The manuscript is structured as follows. In Section 2, we introduce the methodology to account for adsorbed atoms (adatoms) coupled electronically to graphene flakes. In Section 3, the method is applied to two-level adatoms attached to triangular armchair-edged graphene flakes of sizes below 5 nm (Figure 1). We focus particularly on exploring the impact of the electronic coupling channel on optical properties of the adatom, gradually hybridizing with the graphene nanoflake as its distance from the flake is decreased. The results reveal that for distances below 1 nm, this coupling channel may dominate over the optical interactions and qualitatively alter the character of fundamental quantum optical phenomena: the coherent Rabi-type flopping between energy eigenstates or the incoherent decay through spontaneous emission of a photon. Appendices cover the technical details of the model and additional results.

## 2 Model

In this section, we describe the applied model in detail. First, we discuss the free Hamiltonian of electrons in the hybrid system consisting of an adatom located in the vicinity of a graphene nanoflake. The adatom introduces charge inhomogeneity on the flake, inducing strong Coulomb repulsion, which we take into account correcting the tight-binding Hamiltonian within a self-consistent procedure. Next, the Hamiltonian is extended to account for illumination with an external electromagnetic field. The electromagnetic field pushes the electrons away from their equilibrium positions, modifying Coulomb interactions. These perturbations give rise to nonlinear time-dependent terms in the Hamiltonian of the system.

### 2.1 Hamiltonian without external illumination

In graphene, three of the four valence electrons at each carbon atom hybridize to create *sp* ^2^ orbitals, forming *σ*-type bonds responsible for the hexagonal lattice structure. The tight-binding model accounts for electrons introduced by the remaining *p*_*z*_ orbital, oriented perpendicularly to the graphene sheet, and responsible for most of the physical properties of graphene near the Fermi energy [46]. The adatom is represented with a small number of eigenstates that correspond to atomic orbitals, modeled as point sites localized near the flake. The adatom orbitals can directly exchange electrons with selected carbon sites at the flake. It has been shown that particular adatom types on graphene interact with each other through *p*_*z*_ graphene orbitals, which justifies the use of the *π*-electron approximation [50, 51]. The Hamiltonian of a flake of *N* carbon sites and *N*_*a*_ adatom orbitals constructed within the tight-binding approximation reads

(1) $$\begin{array}{ll} H_{\text{TB}} & =H_{\text{flake}}+H_{\text{atom}}+H_{\text{int}}\\ & =-t\overset{N}{\underset{\left\langle l,l^{\prime }\right\rangle }{\sum }}|l〉〈l^{\prime }|+\overset{N_{a}}{\underset{\alpha =1}{\sum }}\epsilon_{\alpha }|\alpha 〉〈\alpha |\\ & \;-\underset{\alpha ,l}{\sum }t_{\alpha ,l}\left(\left|l〉〈\alpha |+|\alpha 〉〈l\right|\right). \end{array}$$

An electron can be exchanged between neighboring carbon atoms at the rate *t*. The symbol ⟨*l*, *l*′⟩ indicates that the summation goes only over nearest neighbor atomic sites *l* and *l*′. The adatom orbitals, labeled by *α*, have energies *ϵ*_*α*_ evaluated with respect to the onsite energy in graphene. The results presented throughout this paper are obtained for adatoms with two levels (a ground state |*g*⟩ and an excited state |*e*⟩) and assume that the adatom introduces only one electron to the system. Electrons can be exchanged between the adatom orbitals and selected flake sites with corresponding tunneling rates *t*_*α*,*l*_. We will focus on armchair-edged graphene flakes, since they have a gap around the Fermi energy, which is interesting for optical effects and also makes them more stable. It is worth noting that armchair-edged nanoflakes do not support edge states. On the contrary, zig–zag terminated flakes support zero-energy edge states of topological nature. These states are located in the middle of the energy gap and would be interleaved on the energy scale with the considered adatom states. This constitutes a more complex case to be investigated in future studies. Adatoms in chains with such topological states have already been investigated in our previous work [45].

For symmetry reasons, three possible positions of adatoms are favorable for the adsorption above the graphene antenna: *top*, *bridge*, and *hollow*. It means that in the nearest-neighbor approximation, the last summation in Eq. (1) can span over one, two or six sites [52]. Independent on the adatom’s type, attaching it to graphene breaks the symmetries of the perfect lattice and, consequently, changes the optical and electronic properties of this material [53]. Throughout this paper, we consider the *top* position of the adatom, meaning that it will be located over and coupled to one particular graphene site. This corresponds to configurations that can be found in covalently functionalized graphene with hydrogen and fluorine adatoms and the hydroxyl group OH and covalently bond adsorbates [47]. Note that in this way, the adatom breaks the sublattice symmetry.

We use the hopping parameter *t* = 2.66 eV between neighboring sites in graphene [54]. The hopping rates between the adatom orbitals and carbon sites *t*_*α*,*l*_ generally depend on the adatom properties and its distance from the flake. We treat them as parameters and leave them unspecified to keep the approach general and characterize the scope of possible physical effects achievable within the model, rather than investigate specific cases. We vary the hopping between 0 and 2*t* which covers most of the reasonable realization scenarios, including atoms, molecules, quantum dots, and defects. Values for *t*_e_, *t*_g_ that are larger than the graphene hopping *t* are feasible for fluorine atoms, OH groups, and some C radicals [47, 50]. On the other hand, values for *t*_e_, *t*_g_ below *t* are feasible for metal adatoms [55]. These values have been verified using more accurate methods, like density functional theory (DFT) [47]. A comparison of results obtained with time dependent DFT and the tight-binding methods has been performed for graphene flakes in particular in Ref. [3].

In Appendix A, we provide the relation of the hopping rates with the distance between the nanoflake and the adatom.

Diagonalization of *H*_TB_ provides a set of energies *E*_*j*_ and eigenstates |*j*⟩. Note that, from now on, we can operate in two different bases to describe the hybrid system: (i) the real-space basis of sites |*l*⟩ and |*α*⟩, which we use to construct the tight-binding Hamiltonian, and (ii) the energy basis of eigenstates |*j*⟩ obtained from the diagonalization of the Hamiltonian. The two bases are related *via* a linear unitary transformation with coefficients *a*_*jL*_:

(2) $$|j〉=\underset{L\in \left\{l,\alpha \right\}}{\sum }a_{jL}|L〉,$$

where we have introduced a joint index *L*, which goes over both flake and adatom sites.

### 2.2 Induced Coulomb repulsion

Given the basic set of eigenstates, we construct the single-particle density matrix as a statistical mixture of density matrices corresponding to eigenstates with energies below or equal to the Fermi energy, according to the Aufbau principle (see Appendix B for details). This density matrix is assumed to represent an average state of one among *N*_e_ electrons. In isolated, undoped graphene flakes, this mixture describes a uniform charge distribution in real space. The presence of the adatom induces a charge nonuniformity on the flake, which due to induced Coulomb repulsion pushes the electrons back towards a uniform distribution *ρ* ^0^. However, the uniform distribution is not the lowest-energy state when the adatom is present. The equilibrium state of the system is a result of a trade-off between two effects, as the energy minimization in the noninteracting system and the Coulomb repulsion balance each other. This equilibrium state *ρ* ^sc^ can be found iteratively in a self-consistency loop, similarly as introduced in Ref. [56]. The self-consistency procedure is described in detail in Appendix B. It additionally provides a self-consistent Hamiltonian *H* ^sc^, a basis set of eigenstates |*j* ^sc^⟩, dressed by the charge-inhomogeneity-induced Coulomb interaction, and the corresponding energies $E_{j}^{\text{sc}}$ . The procedure can be applied for an arbitrary number of *N*_e_ electrons in the system, which allows for taking into account doping with either electrons or holes. In this work, we do not include the exchange energy. In literature, it has been taken into account, e.g., by modification of the Coulomb interaction terms [57]. Similarly, adjusting Coulomb interactions may be used to mimic magnetic effects in certain nanostructures [58], which is also beyond the scope of this work.

**Figure 2:**
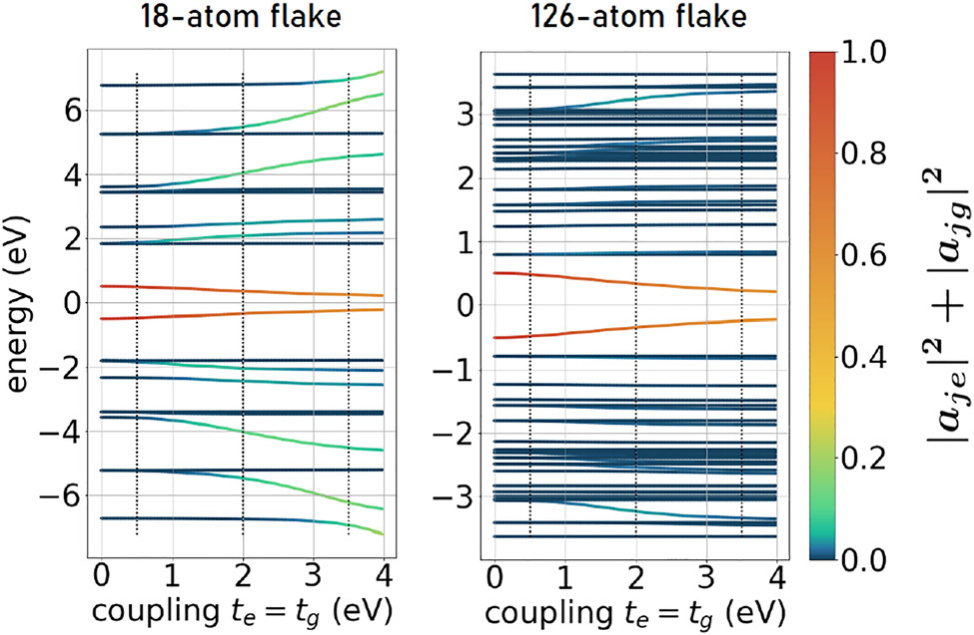
Energy levels of hybrid systems consisting of an armchair triangular graphene flake with 18 atoms (0.71 nm side length, left) and 126 atoms (2.41 nm side length, right) and an adatom with energy levels *E*_e_ = 0.5 eV and *E*_g_ = −0.5 eV (inside the gap around the Fermi energy) located over the left top-most site of the flake. For the larger flake, a selected range of energies around the Fermi energy is presented. The line color indicates the occupation of the adatom sites in a given eigenstate |*j*⟩: |*a*_*j*e_|^2^ + |*a*_*j*g_|^2^, where *e* and *g* denote the excited and ground adatom sites, and *a*_*jL*_ is defined *via* Eq. (2).

**Figure 3:**
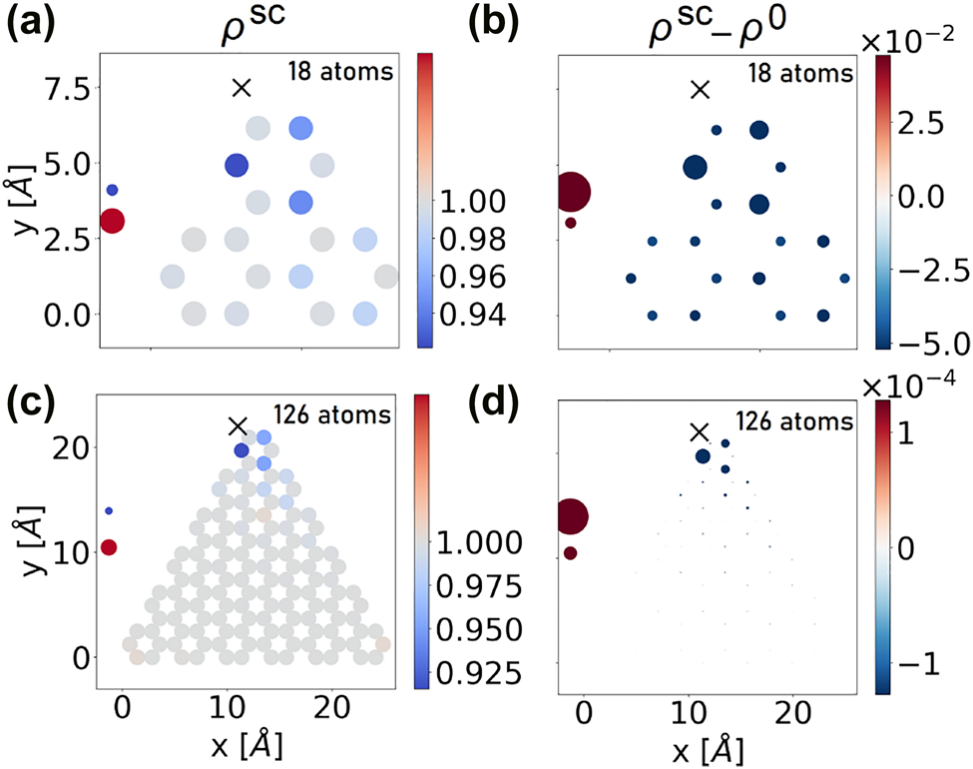
(a and b) Charge distribution on a hybrid system consisting of an armchair triangular graphene flake with 18 atoms and an adatom. The adatom is located near one particular site at a position marked with X and coupled with *t*_e_ = *t*_g_ = 2.0 eV. The adatom level energies lie inside the gap around the Fermi level with *E*_e_ = 0.5 eV and *E*_g_ = −0.5 eV. The plots show the real-space charge distribution due to the self-consistent density matrix *ρ* ^sc^ (a), as well as the difference with respect to the density matrix corresponding to a uniform charge distribution *ρ* ^sc^ − *ρ* ^0^. (b) The occupation of the adatom levels is presented on the left side of the figure (upper dot denotes the excited state, lower dot – the ground state). (c and d) As in (a and b), but for the flake with 126 atoms. On panels (b and d) the area of the spots is proportional to the density matrix modification on a given site ${\left(\rho^{\text{sc}}-\rho^{0}\right)}_{ll}$ .

**Figure 4:**
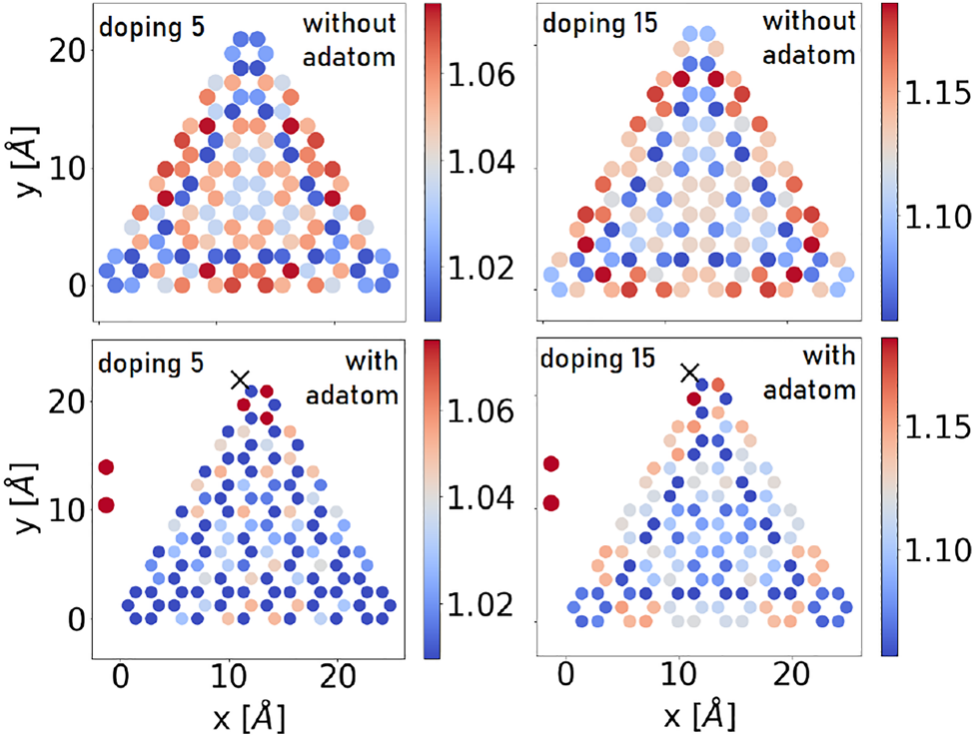
Upper row: Charge distribution due to the self-consistent density matrix *ρ* ^sc^ of an armchair triangular graphene flake of 126 atoms doped with 5 electrons (left) or 15 electrons (right). Lower row: As in the upper one, but with an adatom.

The energy spectra of triangular armchair-edged graphene nanoflakes after the self-consistency procedure are shown in Figure 2. The color of the lines denotes the occupation probability |*a*_*j*g_|^2^ + |*a*_*j*e_|^2^ of the adatom sites in the hybridized eigenstates *j* when the flake and adatom become increasingly coupled. This shows explicitly that the flake eigenstates that mix with the adatom eigenstates most strongly are also the ones that change their energies the most when the hopping parameters *t*_e_ and *t*_g_ are increased. Shifting up or down one of the adatom energy levels breaks the electron–hole symmetry in the energy spectrum of the system, as demonstrated in Appendix C. This has its consequence for the absorption spectra described in Section 2.5, in which the originally degenerate peaks would split, leading to a more complex optical response. The real-space representation of self-consistent density matrices *ρ* ^sc^ corresponding to the charge distributions *N*_e_ *eρ* ^sc^ for triangular graphene flakes of 18 and 126 atoms with adatoms are shown in Figure 3. Here, $N_{e}\rho_{ll}^{\text{sc}}$ is the expectation value of the number of electrons on site *l*. The impact of the adatom on the charge distribution is limited to the small region below 1 nm in size, in practice, including all the carbon atoms of the smaller flake. On the contrary, on a larger flake, the adatom affects the few nearest hexagons [Figure 3(b and d)]. The adatom modifies the symmetry of the structure which is decisive for optical properties. This intuitive result persists in the doped case, as shown exemplarily in Figure 4. With doping, the ground state *ρ* ^sc^ is no longer uniform, and the effect of the adatom on that structure may extend throughout the entire flake. For small doping, the influence from the adatom dominates the charge redistribution effect. But even for higher doping values the effect of the adatom is clearly visible in charge distribution as shown in the case of 15 doping electrons. As a consequence, the optical properties of the flake will be modified, relaxing the selection rules for optically driven transitions.

### 2.3 Including electric field

An electric field **E**(**r**, *t*) can be coupled to the nanoflake through the interaction Hamiltonian

(3) $$H^{\text{int}}\left[\rho \left(t\right),t\right]=H^{\text{sc}}-e\varphi^{\text{ext}}\left(t\right)-e\varphi^{\text{ind}}\left[\rho \left(t\right)\right],$$

where *ρ*(*t*) is the time-dependent density matrix of the system evaluated based on the master Eq. (7) described in details in Section 2.4. The external potential *φ* ^ext^(*t*) is given by

(4) $$\begin{array}{ll} \varphi^{\text{ext}}\left(t\right) & =-\underset{L}{\sum }r_{L}\cdot E\left(r_{L},t\right)|L〉〈L|\\ & \;+r_{eg}\cdot E\left(r_{0},t\right)\left(\left|e〉〈g|+|g〉〈e\right|\right), \end{array}$$

where **r**_*L*_ is the position of the given site and **r**_0_ – of the adatom, **d**_eg_ = *e* **r**_eg_ is the transition element of the dipole moment operator evaluated between the adatom states. In our model, the flake is described by one orbital per site. Therefore, *φ* ^ext^ is diagonal in the site basis, except for the elements which couple the adatom states. A transformation to the energy basis will then reveal nonzero off-diagonal elements responsible for electromagnetic coupling of the hybrid structure’s eigenstates. On the contrary, the internal geometrical structure of the adatom is beyond the level of approximation applied here, so it is explicitly included *via* the transition dipole moment **d**_eg_.

As the electric field moves the electrons away from their equilibrium positions, Coulomb repulsion within the electron cloud arises, accounted for by the field-induced potential

(5) $$\begin{array}{ll} \varphi^{\text{ind}}\left[\rho \left(t\right)\right] & =-eN_{e}|L〉〈L|\underset{L^{\prime }=\left\{l^{\prime },\alpha^{\prime }\right\}}{\sum }v_{LL^{\prime }}\left(\rho_{L^{\prime }L^{\prime }}\left(t\right)-\rho_{L^{\prime }L^{\prime }}^{\text{sc}}\right), \end{array}$$

where the Coulomb elements *v*_*ll*_′ have been evaluated in Ref. [59] and are listed in Appendix D.

The oscillating induced charge density generates an induced electric field [60] which reads as:

(6) $$E_{\text{ind}}\left(r,t\right)=\frac{1}{4\pi \epsilon_{0}}\underset{l}{\sum }\frac{Q_{l}\left(t\right)\left(r_{l}-r\right)}{|r_{l}-r{|}^{3}}$$

where $Q_{l}\left(t\right)=N_{e}e\left(\rho_{ll}\left(t\right)-\rho_{ll}^{\text{sc}}\right)$ is the charge induced on the *l*th site at time *t* and *ϵ*_0_ denotes the vacuum permittivity. In all our calculations, the electric field **E**(**r**, *t*) in Eq. (4) is the sum of an external electric field **E**_ext_(**r**, *t*) and the induced electric field **E**_ind_(**r**, *t*).

**Figure 5:**
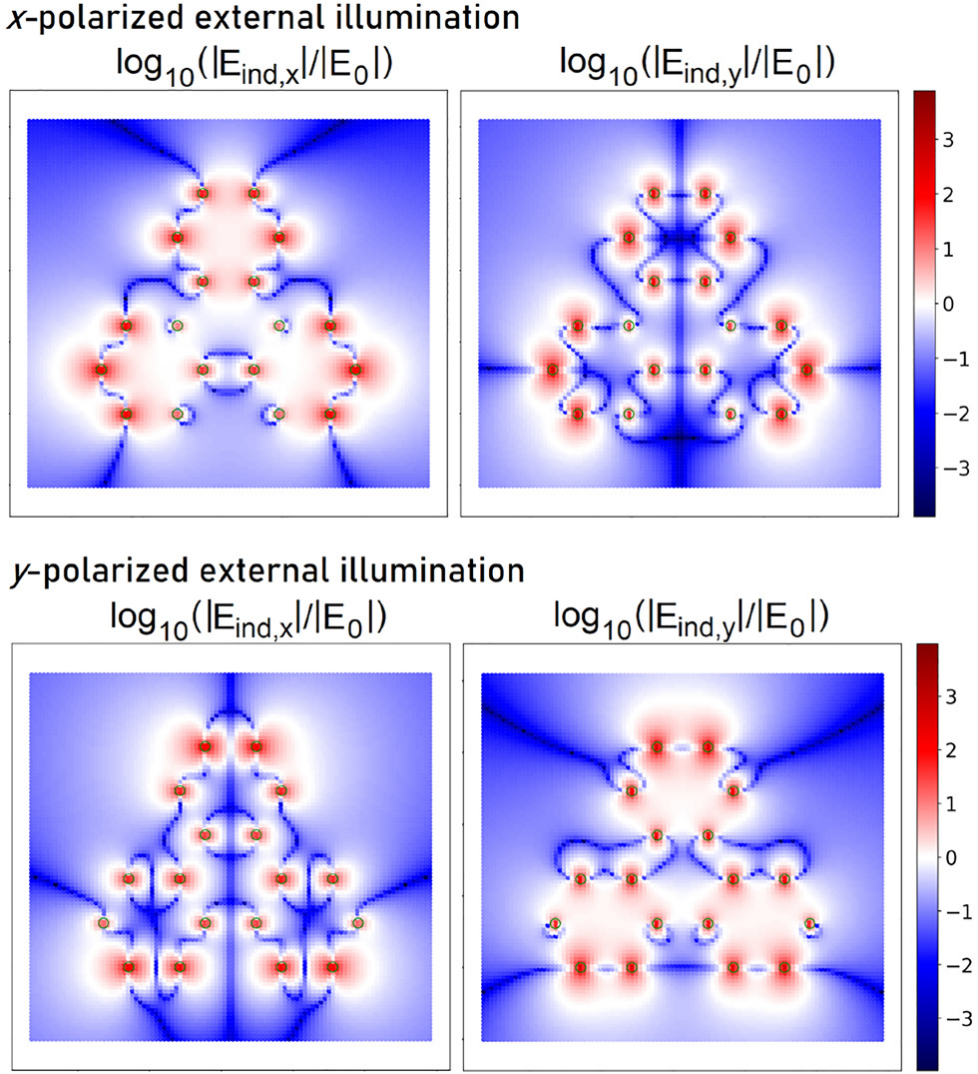
Distribution of the *x*-components (left) and *y*-components (right) of the electric field that is induced around an 18-atom graphene nanoflake due to an external illumination polarized either in *x*-direction (upper panels) or *y*-direction (lower panels).

The induced field distribution near an 18-atom flake subject to a plane-wave illumination resonant with the adatom transition frequency of 1 eV is illustrated in Figure 5. In the blue regions, the induced field amplitude is lower than that of the illuminating field, while the red color indicates regions where the induced field dominates over the external one. Apparently, higher-order interactions could still be neglected for adatom distances of the order of carbon–carbon distance in graphene. Dipolar and quadrupolar coupling mechanisms may become comparable at short distances corresponding to dark red areas in Figure 5. Higher-order interactions would also be increased in even stronger fields or for illumination frequencies tuned to flake resonances.

### 2.4 Dynamics

The dynamics of the system is described with the single-electron master equation

(7) $$\frac{\partial }{\partial t}\rho \left(t\right)=-\frac{i}{\hbar }\left[H\left(\rho \left(t\right),t\right),\rho \left(t\right)\right]-D\left[\rho \left(t\right)\right].$$

The Hermitian Hamiltonian described in previous sections accounts for the reciprocal processes in the system, with the nonlinearity related to the inclusion of the Coulomb interactions. The phenomenological damping term reads as

(8) $$D\left[\rho \left(t\right)\right]=\frac{1}{2\tau }\left(\rho \left(t\right)-\rho^{\text{stat}}\right).$$

where *τ* is the coherence lifetime known from experiments in bulk graphene. It characterizes the dissipation of the system adiabatically moving towards a predefined stationary state *ρ* ^stat^. In practice, we choose *ρ* ^stat^ = *ρ* ^sc^ arising from the self-consistency procedure, so that the dissipation forces the system back into its equilibrium state. In general, different lifetimes could be assigned to specific elements of the density matrix, in particular, when considering the adatom and the flake. Such a modification would not make a significant impact in the cases investigated below.

### 2.5 Absorption spectra

**Figure 6:**
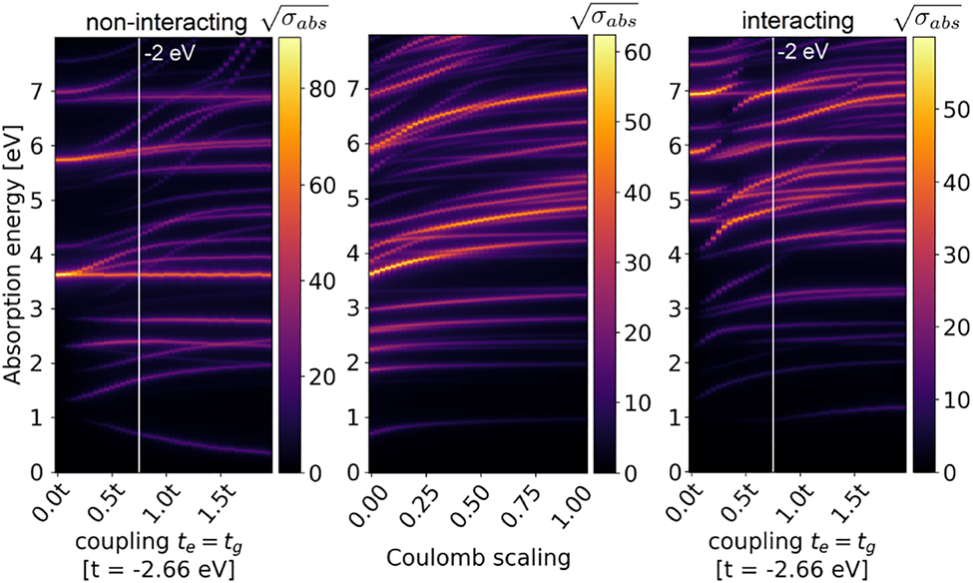
Optical absorption spectra for an 18-atom flake with an adatom having energy levels at *E*_e_ = 0.5 eV, *E*_g_ = −0.5 eV. Left panel: Noninteracting spectra. Middle panel: Dependence of the spectrum on the Coulomb interaction strength for *t*_e_ = *t*_g_ = −2 eV. Right: Interacting absorption spectra.

Square roots of the optical absorption spectra for the 18-atom flake with the adatom are shown in Figure 6 (see the calculation method in Appendix E). The noninteracting absorption spectrum in the left panel corresponds directly to the tight-binding energy spectra from Figure 2. The spectra of a bare flake correspond to *t*_e_, *t*_g_ = 0, while the impact of the adatom is revealed for nonzero hopping rates. The adatom introduces new low-energy resonances that arise from transitions involving its own states. Additionally, it influences the frequency and strengths of other transitions. Some transitions missing in the spectrum of a bare flake appear at presence of the adatom, which is caused by the flake symmetry breaking and the corresponding modification of selection rules. As evident from the energy spectra in Figure 2, only selected flake eigenstates couple to the adatom. This results in the splitting of resonances already visible in the bare flake spectrum. These observations are in line with our previous results [45] for linear atom chains with adatoms. The middle panel shows the dependence of the absorption spectra on the Coulomb interaction strength for fixed adatom–flake hopping rates *t*_e_ = *t*_g_ = −2 eV. The Coulomb scaling parameter on the horizontal axis multiplies the induced Coulomb interactions. The figure explains how the Coulomb interaction shifts optical resonance positions of the system and reveals the connection between the noninteracting and interacting absorption spectra. The right panel shows the interacting absorption spectrum including the Coulomb interaction with the scaling parameter equal to 1. Despite the fact that some resonances are significantly shifted, the conclusions drawn about the adatom impact in the noninteracting case remain valid.

## 3 Impact on basic quantum-optical phenomena

Here, we apply the model to demonstrate the impact of the electron exchange between adsorbed atoms and nanoflakes on fundamental quantum-optical phenomena: the coherent Rabi oscillations and the incoherent spontaneous emission in a two-level system. We find a significant influence of the electron exchange on the character of the coherent dynamics: For relatively small hopping rates *t*_e,g_ < *t* and atom–flake distances in the order of 0.5 nm or longer, the frequency of the Rabi oscillations between a pair of hybridized eigenstates is modified in accordance with the modulation of the transition dipole between them. For larger coupling strengths, additional states may significantly contribute to the energy exchange. In parallel, the induced Coulomb interactions effectively introduce time-dependent detuning, eventually blurring the oscillatory character of the dynamics completely. The model also reveals that the impact of the electronic coupling channel on the Purcell decay rate enhancement may be significant on rather short distances, i.e., below 1 nm. This is in agreement with DFT-based studies of quantum emitters in close proximity to metal nanoparticles [4]. Note that all distances are estimated according to the model described in Appendix A.

### 3.1 Modification of Rabi oscillations

As the generic model of quantum optics, the two-level atomic system subject to external illumination undergoes sinusoidal Rabi oscillations of population between its ground and excited states. The population oscillation amplitude is equal to 1 in the case of resonance, and the Rabi oscillation frequency reads as

(9) $$\Omega_{\text{res}}=E\cdot d_{eg}/\hbar ,$$

where **E** is the field amplitude the system is subject to. For moderately detuned fields, in the range of applicability of the rotating wave approximation, this picture holds with the oscillation amplitude reduced and the Rabi frequency increased to [61]

(10) $$\Omega =\sqrt{\Omega_{\text{res}}^{2}+\Delta^{2}},$$

where Δ is the detuning between the continuous wave illumination frequency and the transition frequency.

Below, we study how coupling the two-level emitter to a graphene nanoflake affects this phenomenon. We study the evolution of the system by solving the master Eq. (7) and find oscillatory behavior in the population dynamics of the HOMO and LUMO states of the coupled system. In this case, the system is subject to a field that includes the external illumination in the form of a plane wave modeled in the quasistatic limit $E_{\text{ext}}\left(r,t\right)=E_{\text{ext}}\left(t\right)$ and the induced field $E_{\text{ind}}\left(r,t\right)$ evaluated according to Eq. (6). The induced field is strongly position-dependent and involves both *x* and *y* polarizations (Figure 5). When the resulting dynamics pertains oscillatory character, this leads to a corresponding position and polarization dependence of Rabi frequency as suggested by Eq. (9). For particular adatom positions, the Rabi oscillation frequency can be strongly enhanced (reddish regions in Figure 5). From the same figure it follows that it would be possible to drive transitions of a system whose dipole moment is oriented perpendicularly to the polarization of the incoming beam. This possibility arises due to the existence of all polarizations in the induced near field and is not present in free space.

**Figure 7:**
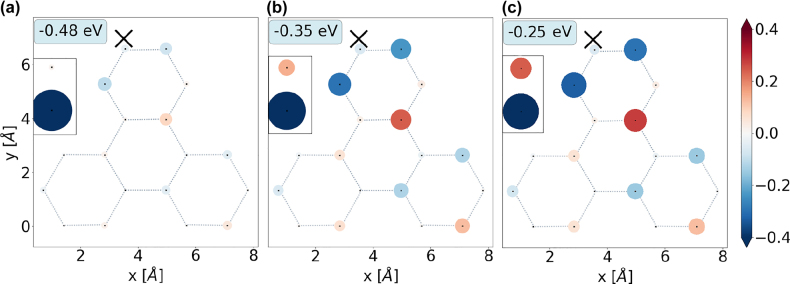
Real-space distribution of the HOMO state in a triangular 18-atom flake with an adatom attached near the tip of the flake at coordinates (3, 7.5) Å with a coupling strength of (a) *t*_e_ = *t*_g_ = 0.5 eV, (b) *t*_e_ = *t*_g_ = 2.0 eV, (c) *t*_e_ = *t*_g_ = 3.5 eV. The black mark X denotes the site to which the adatom is coupled. The area of the spots is proportional to the HOMO contribution to the population of a given site in real space.

We now focus on hybrid systems that consist of an undoped armchair-edged triangular nanoflake with 18 atoms (side length 0.71 nm) or 126 atoms (side length 2.41 nm) and a two-level adatom with energies 0.5 eV and −0.5 eV, characterized with a dipole moment *d*_eg_ = 7.5 D. The rather large value of the dipole moment assures relatively fast Rabi oscillations and, therefore, the demonstration of the effect requires relatively short simulation times. Moreover, for the chosen value of the external field, this dipole moment results in Rabi oscillations on a similar time-scale as the dissipation processes in bulk graphene. Here, we only consider adatom levels which are located in the energy gap around the Fermi energy. Moreover, we assume for simplicity that both adatom levels are coupled equally strongly to a carbon atom at the corner of the graphene flake, i.e., *t*_e_ = *t*_g_ for all simulations. We investigate hopping parameters *t*_e_ and *t*_g_ up to 3.5 eV, which corresponds to distances in the order of Angstroms and larger (see the model in Appendix A). The external field has an amplitude of $0.05\;\frac{\text{V}}{Å}$ and is polarized along the *y*-axis, as defined in Figure 7. Damping effects are neglected by setting D(ρ)=0 in Eq. (7).

We first analyze the dynamics neglecting the electron–electron interactions in the system, i.e., setting *φ*_ind_ = 0 in Eq. (3). This allows us to understand the basic process that occurs as the adatom gets coupled to the flake with increasing strength *t*_e,g_ – the change of frequency of the Rabi oscillations between a pair of states.

**Figure 8:**
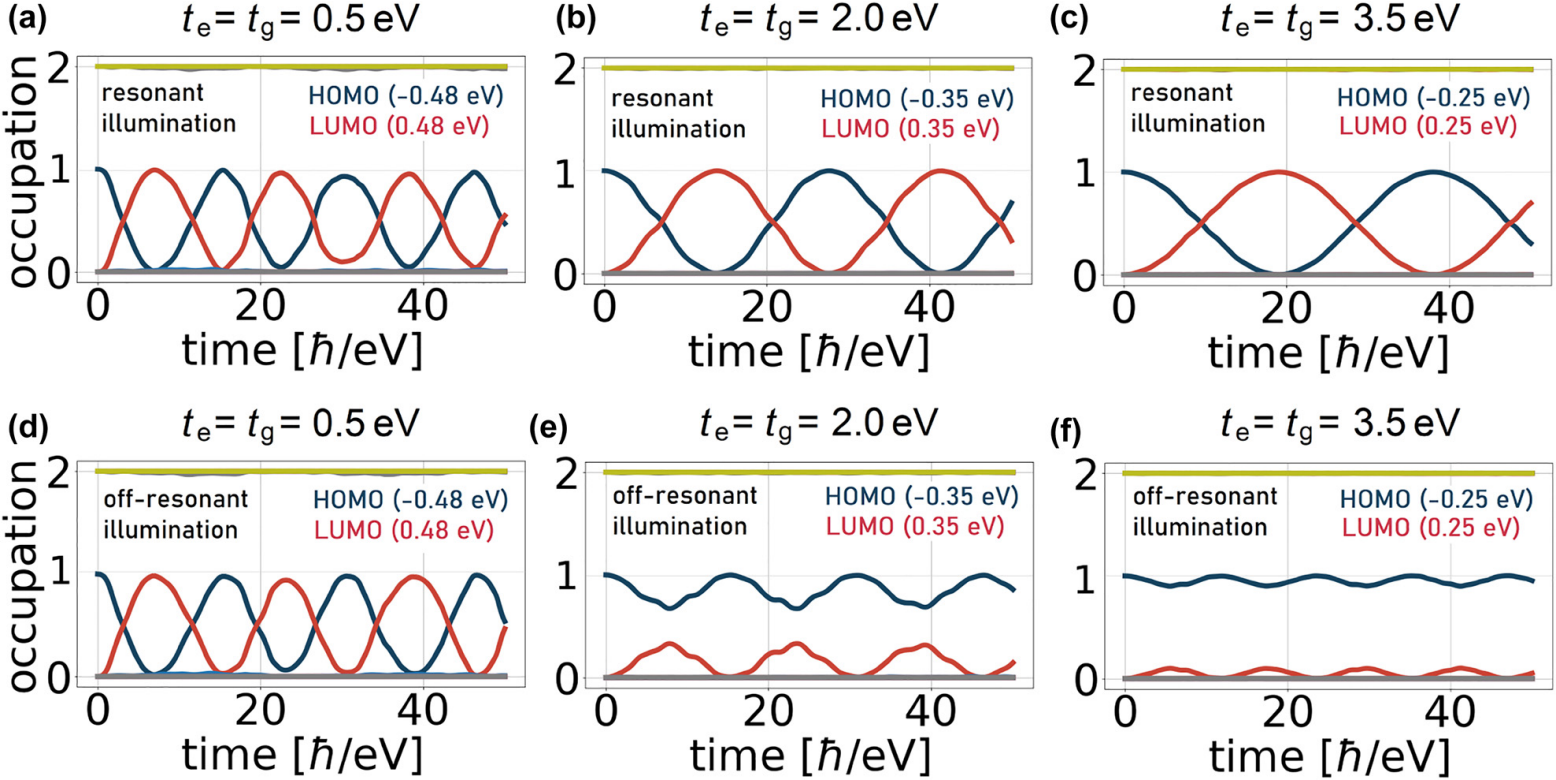
Occupation dynamics with evident Rabi oscillations between the HOMO and LUMO states in the 18-atom flake with an adatom. Here, the Coulomb interaction is not included. In the “resonant illumination” case (a–c), the illumination frequency is always resonant to the HOMO–LUMO transition frequency. In the “off-resonant illumination” case (d–f), the illumination frequency is fixed and equal to 1 eV in each case.

In the absence of coupling, the adatom states |*g*⟩ and |*e*⟩ fit in the energy gap of the graphene flake (Figure 2). As the coupling strengths between the subsystems increase, the orbitals hybridize and shift in energies. In particular, the HOMO and LUMO states become localized on both subsystems with the dominant contributions originating from the adatom and a small part coming from the flake. There is no energy crossing involving the pair of orbitals, which originate from the adatom, that retain their HOMO/LUMO character. We illuminate the system with a monochromatic field resonant with the LUMO – HOMO transition energy, which now depends on *t*_e,g_. For the set of parameters listed above, we find that the stronger the adatom is coupled to the graphene flake, the lower the frequency of Rabi oscillations it exhibits [Figure 8a–c].1The density matrix is normalized such that the occupancy of each level can go up to two. This can be explained by the fact that the transition dipole moment of an isolated adatom *d*_eg_ has been set to a relatively large value. On the other hand, the dipole moment operator elements $d_{jj^{\prime }}=N_{e}e|\left\langle j|\hat{r}|j^{\prime }\right\rangle |$ of isolated flakes considered here are lower and up to 5 D. In the coupled case *t*_e,g_ ≠ 0, the states originally corresponding to the adatom acquire components localized on the flake [Figure 7] and vice versa. The corresponding transition dipole moment elements reflect this mixing effect: The dipole moment **d**_*jj* ^′^_ between the *j* = LUMO and the *j*′ = HOMO states is decreased with respect to the original dipole moment in the adatom **d**_eg_ due to the admixture of the flake component. This leads to a reduction of the Rabi frequency as suggested by formula (9).

Next, we fix the illumination frequency to be constant and equal to the frequency difference (*ϵ*_e_ − *ϵ*_g_)/ℏ of an uncoupled two-level system. As the adatom is increasingly coupled to the nanoflake, the energy structure of the system is modified so that, in this case, detuned behaviour is expected – an increased Rabi frequency and a lowered amplitude of oscillations [61]. This is shown in Figure 8d–f.

**Figure 9:**
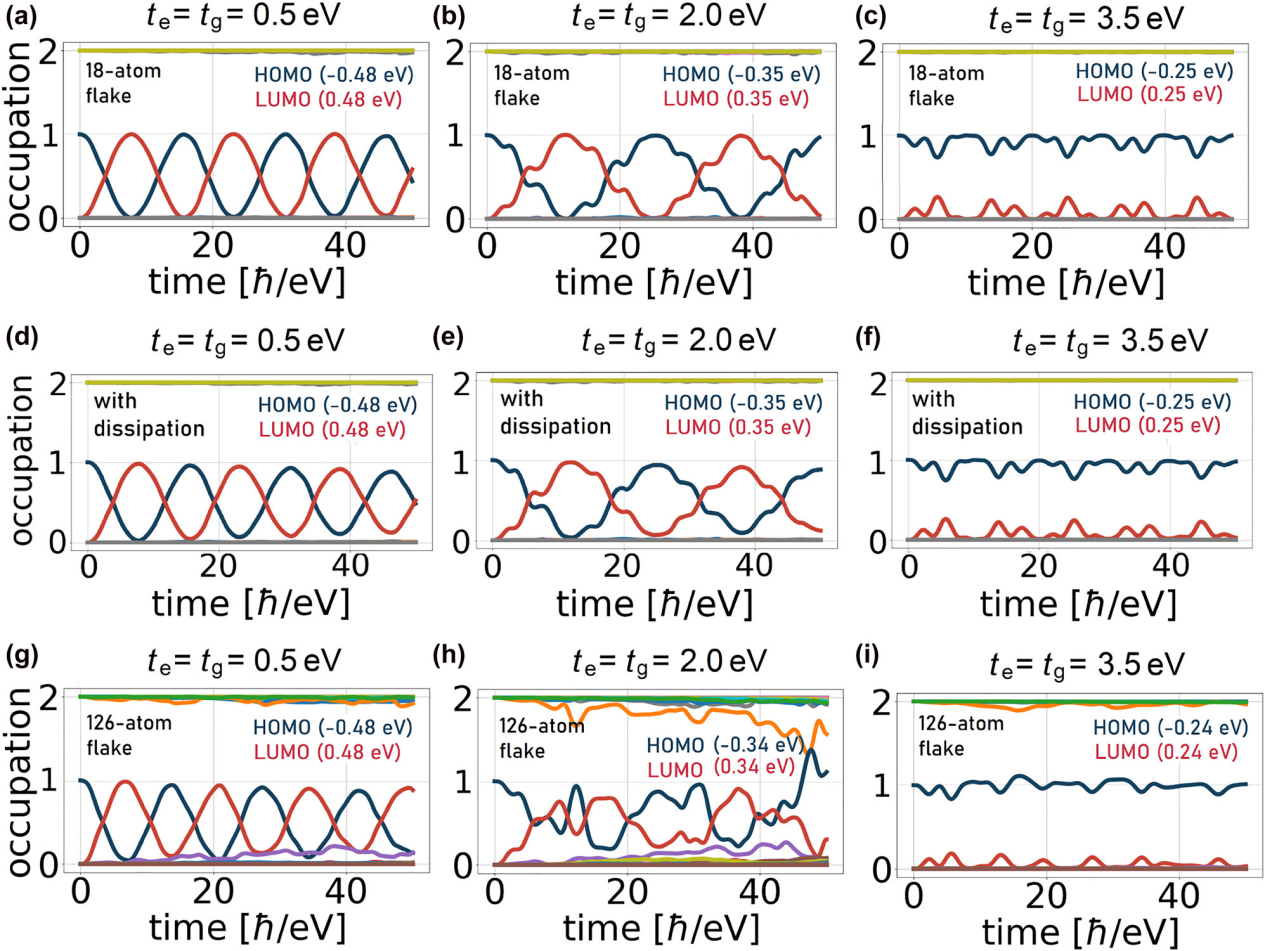
Occupation dynamics for the hybrid system illuminated at a frequency resonant to the HOMO–LUMO transition. This time, the Coulomb interaction is included. (a–c) 18-atom flake without dissipation, (d–f) 18-atom flake with dissipation (ℏ*τ* ^−1^ = 10 meV), (g–i) 126-atom flake without dissipation. In panels g–i, the orange line corresponds to the occupation of the HOMO−2 state and the purple line to the LUMO+2 state, whose energies ∓0.82 eV happen to be separated from the HOMO/LUMO states by a difference almost resonant with the illumination frequency.

Finally, we include the electron–electron interaction potential $\varphi^{\text{ind}}\left[\rho \left(t\right)\right]$ given by Eq. (5) and look again at the time evolution of the hybrid system (Figure 9a–c). The system is illuminated with the HOMO–LUMO transition frequencies evaluated self-consistently according to the procedure in Appendix B. On top of the Rabi frequency modification due to hybridization, we now note additionally a relatively fast modulation and a detuning effect that arise from the time-dependent Coulomb potential $e\varphi^{\text{ind}}\left[\rho \left(t\right)\right]$ , which adds to the system Hamiltonian and modulates the energy eigenvalues locally in time. We also repeat the same calculation including dissipation with a relaxation time *τ* determined from ℏ*τ* ^−1^ = 10 meV, which is a realistic value for graphene. The decay rate is rather slow compared to the timescale of Rabi oscillations, such that multiple cycles of the process can be clearly observed, as shown in Figure 9d–f.

The results presented above concern the adatom positioned at a distance similar to the carbon–carbon distance in graphene. For the applied illumination frequency, the induced field has a moderate effect on the dynamics. In doped flakes, we anticipate a stronger effect exploiting plasmonic enhancement of light–matter interaction strength resulting in Rabi oscillations at considerably higher frequencies.

**Figure 10:**
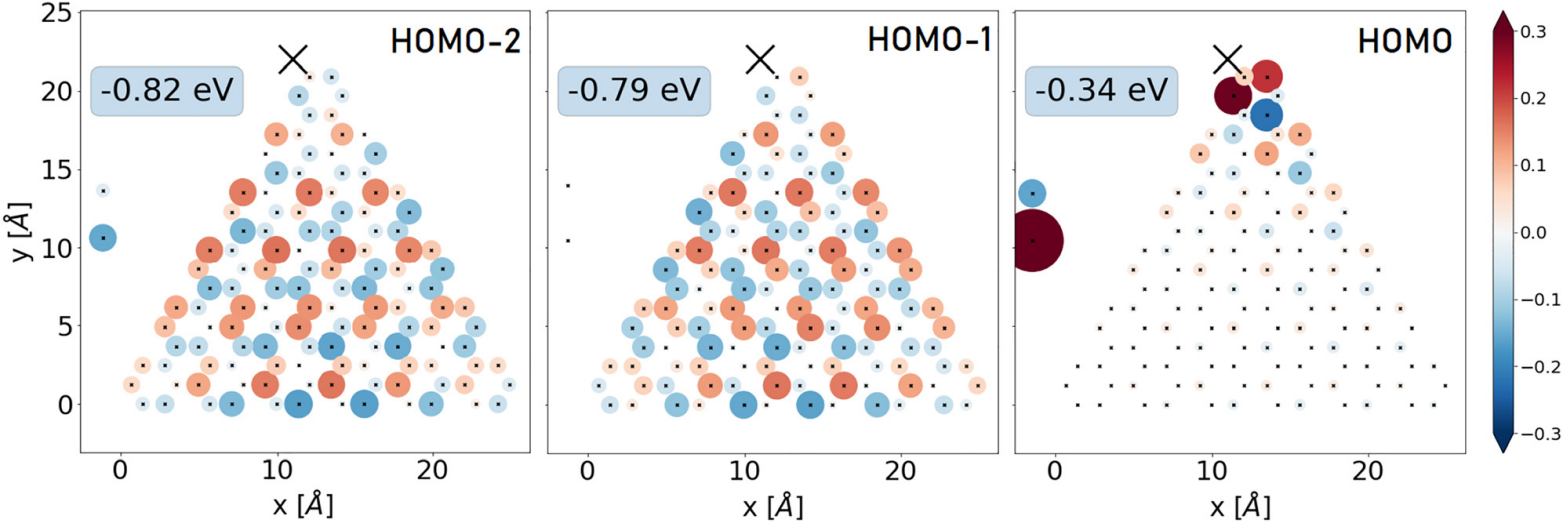
Real-space distribution of the HOMO, HOMO−1 and HOMO−2 states in a 126-atom flake with the adatom attached with a coupling strength of *t*_e_ = *t*_g_ = 2.0 eV near the tip of the flake at coordinates (11, 22) Å. The black mark X denotes the location of the adatom in the *XY* plane.

These effects appear in a similar form in larger nanoflakes (Figure 9g–i). However, due to the increased density of states in larger structures, it is generally more likely that additional states originating from the flake actively contribute to the energy exchange as transitions involving these states get close to resonance with the frequency of the illuminating field. This is illustrated in Figure 9h for *t*_e,g_ = 2.0 eV, where the orange and violet lines indicate transitions involving the states with energies ±0.82 eV, being the LUMO+2 and HOMO−2 states at approximately twice the value of the HOMO/LUMO energies. Here, the coupling with the adatom has lifted the degeneracy of these states with the LUMO+1 and HOMO−1 states, which remain uncoupled to the adatom. As a result, the symmetry of states that hybridize with the adatom is broken, as shown in Figure 10 for the HOMO, HOMO−1, and HOMO−2 states, which play the most important roles in interactions with light. The symmetry breaking modifies the selection rules allowing for optical transitions to states of arbitrary symmetry in general.

### 3.2 Spontaneous emission

In an isolated atom, energy dissipation occurs radiatively through spontaneous emission [61]. A lot of efforts have been devoted to exploit traditional or nanophotonic cavities to control the emission rate *via* the so-called Purcell effect [28, 62, 63]. The control is possible by adjusting the geometrical parameters of the cavity to modify the density of electromagnetic states that can be accounted for with the electromagnetic Green’s tensor associated with the cavity [64]. The formalism has been applied to plasmonic nanoantennas playing the role of a cavity as well [65–67]. Here, we discuss an example of the same phenomenon near a graphene nanoflake, where, besides the optical coupling channel between the adatom and the flake, electron tunneling effects occur and may modify the result.

#### 3.2.1 Green’s tensor method

The atomic spontaneous emission rate can be modified in the presence of cavities or scatterers. This effect is *optical* in its origin and can be quantified in the electromagnetic Green’s tensor formalism [65]:

(11) $$\Gamma =\frac{2\omega_{jj^{\prime }}^{2}}{\hbar \epsilon_{0}c^{2}}d_{jj^{\prime }}\text{Im}\;G\left(r_{0},r_{0},\omega_{jj^{\prime }}\right)d_{jj^{\prime }},$$

where *ω*_*jj*′_ is the transition frequency between a pair of states and *c* is the vacuum speed of light. We set *j* = L and *j*′ = H, corresponding to the LUMO and HOMO states of the coupled system, dominated by the atomic component (Figure 2). This allows us to work in the approximation that the dipole is mainly located at the adatom position.

By definition, in the linear response regime the Green’s tensor $G\left(r,r^{\prime },\omega \right)$ connects a dipolar source **d**(**r**′, *ω*) at position **r**′, oscillating at the frequency *ω*, with the electric field at position **r** given by:

(12) $$E\left(r,\omega \right)=\frac{\omega^{2}}{\epsilon_{0}c^{2}}G\left(r,r^{\prime },\omega \right)d\left(r^{\prime },\omega \right).$$

The tensor **G** = **G**_*H*_ + **G**_*S*_ can be decomposed into the homogeneous part **G**_*H*_ that here represents the response in free space, and the scattered part **G**_*S*_ that accounts for the presence of scatterers, here – the flake. Consequently, the emission rate can be decomposed into its homogeneous and scattered contributions Γ = Γ_*H*_ + Γ_*S*_.

#### 3.2.2 Influence of electron tunneling

For the homogeneous component that neglects the presence of scatterers we find in the limit of **r**′ → **r** that $ImG_{H}\left(r,r,\omega \right)=\frac{\omega }{6\pi c}$ [65, 66] and the expression for the homogeneous part of the emission rate in Eq. (11) simplifies to the Weisskopf–Wigner formula. Even though the homogeneous part does not account for the *optical* interaction between the adatom and the flake, it can be influenced by *electron tunneling*, i.e., hopping between the adatom and the flake. This is due to the dependence of the transition dipole moment **d**_*jj* ^′^_ and frequency *ω*_*jj* ^′^_ on the values of the hopping rates *t*_e,g_, corresponding to the adatom distance to the nearest carbon site on the flake.

**Figure 11:**
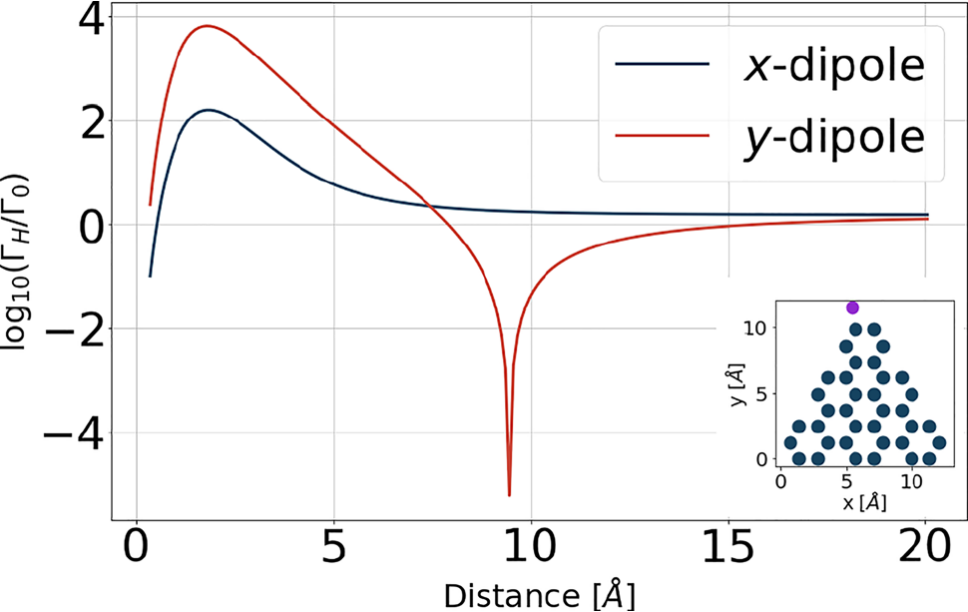
Influence of electron tunneling effects on the spontaneous emission rate between LUMO and HOMO states of a hybrid system as a function of the distance between the adatom and the closest carbon atom in the nanoflake. The considered nanoflake is a triangular armchair-edged graphene flake with 36 atoms and the adatom has energies ±1 eV. The adatom is attached in the upper-left corner of the nanoflake and is marked in the subfigure as a purple dot, and moved away from the nanoflake in the *y*-direction. The dipole moment in the uncoupled adatom is set to *d*_eg_ = 0.01 eÅ ≈ 0.05 D to ensure that we stay in the linear response regime and is oriented in the *x*-direction or *y*-direction. The distance has been evaluated based on the coupling strengths *t*_e_ and *t*_g_ according to the formula in Appendix A (see Figure 13) with *β* = 1.

The dependence of the resulting spontaneous emission rate on the distance is presented in Figure 11, where the result is normalized to the free-space rate of a decoupled atom $\Gamma_{0}=\frac{\omega_{eg}^{3}|d_{eg}{|}^{2}}{3\pi \epsilon_{0}\hbar c^{3}}$ . In fact, it is the hopping rate *t*_e,g_ that enters the calculations rather than the distance directly, while their relation is given in Appendix A with *β* = 1. However, for easier comparison with the following results we plot the emission rates as a function of the distance. For the *y*-orientation of the dipole we note the dip centered at the distance of about 9 Å. It arises due to a destructive interference of two dipole moment contributions: the transition dipole of the adatom *d*_eg_ and the dipole moment component arising on the flake. The two contributions cancel each other at the mentioned distance. This effect depends on the orientation of the dipole.

#### 3.2.3 Influence of optical coupling

To evaluate the scattered Green’s tensor based on Eq. (12), we illuminate the nanostructure with a dipolar electric field from a source of amplitude and polarization in accordance with **d**_LH_ located at the position of the adatom **r**_0_. We propagate the entire system in time and calculate the scattered part of the field **E**_ind_(**r**_0_, *t*) at the adatom’s position using Eq. (6). The electric field in Eq. (12) is then the Fourier component of **E**_ind_(**r**_0_, *t*) corresponding to the transition frequency, and only its imaginary part is relevant for the spontaneous emission rate in Eq. (11).

**Figure 12:**
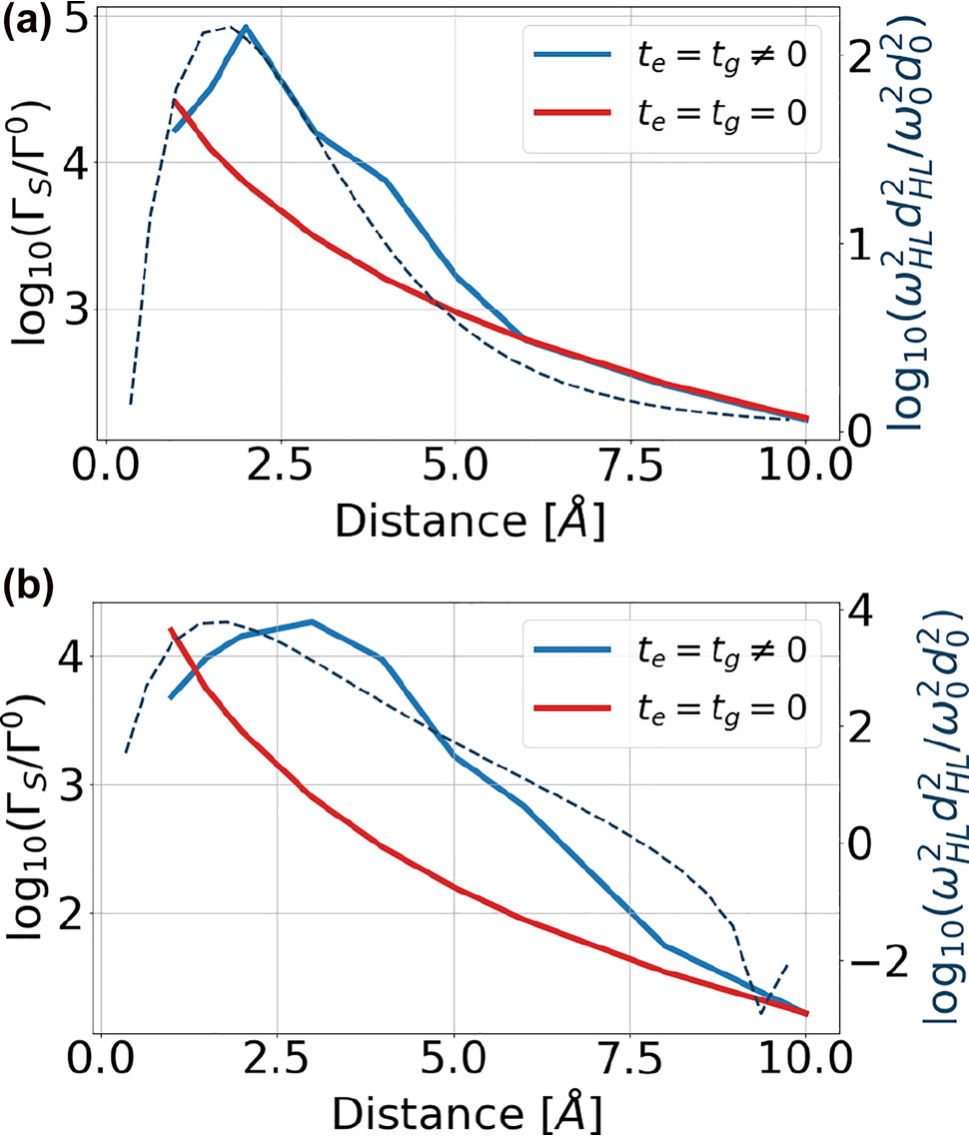
Dependence of the spontaneous emission rate on the distance between the nanoflake and the adatom for the electronically decoupled case with *t*_e,g_ = 0 (red solid line) and including the electronic coupling mechanism with *t*_e,g_ (blue solid line) being functions of distance, for the adatom transition dipole moment oriented in the *x* (a) or *y* direction (b). The considered nanoflake is a triangular armchair-edged graphene flake with 36 atoms and the adatom has energies ±1 eV. The dipole moment in the uncoupled adatom is set to *d*_eg_ = 0.01 eÅ ≈ 0.05 D. The dashed line presents an analytical prediction of how the resonant frequency and the HOMO–LUMO transition dipole moment in the coupled system changes depending on the distance between the nanoflake and the adatom. The expression in the *y*-label contains squared values of frequency and dipole moment such that they coincide with Eq. (11).

The resulting dependence of the spontaneous emission rate Γ_S_ on the distance between the nanoflake and adatom is presented in Figure 12 for two dipole orientations in the *x*- or *y*-direction. The adatom dipole moment has been set to *d*_eg_ = 0.01 eÅ ≈ 0.05 D to assure the linear response regime. For the hopping rate *t*_e,g_ = 0, the adatom is *electronically decoupled* from the flake, and the spontaneous emission enhancement originates only from the *optical* coupling mechanism. This enhancement is shown with red solid lines as a function of adatom distance from the nearest carbon site. For the *x*-orientation of the dipole, the optical coupling with the flake is much more efficient and the calculated transition rates are 1–2 orders of magnitude larger. Please note that since only the scattered component of the Green’s tensor is taken into account in this calculation, the limiting value of the transition rate at large distances is zero for both orientations. At distances in the order of a few Å, the scattered part of the emission rate Γ_S_ is by far the dominant contribution, overcoming the homogeneous one evaluated in the previous section by more than 3 orders of magnitude. The value of Γ_S_ drops below the free-space value Γ_0_ for distances on the order of 15–40 nm, depending on the dipole orientation.

#### 3.2.4 Combined influence of optical coupling and electron tunneling

For short distances below 10 Å, for which the electron tunneling effect is relevant, we repeat the calculation of Γ_S_ including the electron tunneling effect with *t*_e,g_ ≠ 0, whose exact value is related to the distance. Results are shown in Figure 12 with blue solid lines. Below 10 Å, the tunneling effect becomes relevant and is responsible for increased values of the transition dipole moment |**d**_LH_| between the LUMO and HOMO states. Consequently, the transition rate is increased according to Eq. (11). The quenching effect at very short distances is related to suppression of the LUMO to HOMO transition frequency due to hybridization and tunneling. This interpretation is in accordance with the dashed lines in Figure 12 showing the distance dependence of the quantity $\omega_{LH}^{2}|d_{LH}{|}^{2}$ , which appears in Eq. (11), but neglecting the electromagnetic field enhancement covered by the Green’s tensor.

## 4 Summary

In this work, we have introduced a framework for modelling graphene nanoflakes with adatoms, that combines a single-particle tight-binding approach to model the electronic properties with a master equation framework to model the dynamics of the system. The many-body character of the system is accounted for *via* a nonlinear, density-matrix-dependent correction of the Hamiltonian that describes Coulomb electron–electron interactions induced by an external electromagnetic field. As a result, within one framework, we can capture two distinct coupling mechanisms of the subsystems, i.e., the optical interaction and the electron hopping, which are usually studied separately. The favorable linear scaling of the Hilbert space size with the number of atoms allows to treat relatively large systems of hundreds of particles.

The model has been applied to example cases focusing on adatoms described with a two-level model with energies inside the HOMO–LUMO gap of the flake. Then, due to electron hopping, the flake and the adatom form a hybrid system. For moderate coupling strengths, the HOMO and LUMO states, decisive for optical properties of the system, spread across both subsystems that, consequentially, both impact these properties. In particular, the increased delocalization of the HOMO/LUMO wavefunctions is responsible for a hopping- (or distance-) dependent suppression of the HOMO–LUMO energy gap. Moreover, the adatom breaks the symmetry of the eigenstates modifying the selection rules defining the optically allowed transitions.

Finally, we have studied the impact of the electron tunneling on phenomena routinely discussed in quantum optics of two-level systems. The investigated examples highlight the role of Coulomb interactions that, in general, play the dominant role in the dynamics, blurring the characteristic oscillatory behaviour associated with the Rabi flopping. At short distances below 1 nm the electron hopping may lead to a strong modification of the Purcell-enhanced spontaneous emission, for which the model predicts quenching related in particular to the suppression of the HOMO–LUMO transition frequency. These examples demonstrate that the cavity-QED based approaches, often employed when discussing quantum emitters optically coupled to plasmonic nanoantennas, may need to be revised for graphene-based nanostructures that require very short quantum emitter (adatom) distances below 1 nm, at which electron tunneling becomes relevant.

## Figures and Tables

**Figure 13:**
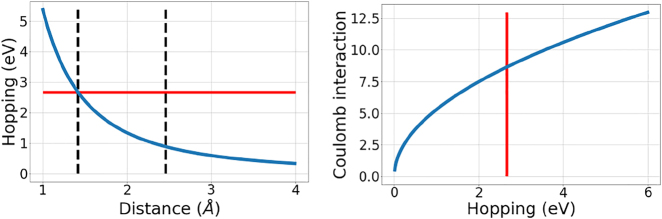
Left: Hopping parameter *t*_*α*_ between the adatom and the nearest carbon atom site as a function of the distance between them. Right: Coulomb interaction as a function of the hopping parameter. The black dashed lines are located at distances related to the graphene structure: the distance between nearest atoms in graphene $\left(2.46\;Å/\sqrt{3}\approx 1.42\;Å\right)$ and the lattice constant (smallest distance between two atoms that belong to the same sublattice, at 2.46 Å). The red lines are located at the hopping value for pure graphene: *t* = 2.66 eV.

**Figure 14:**
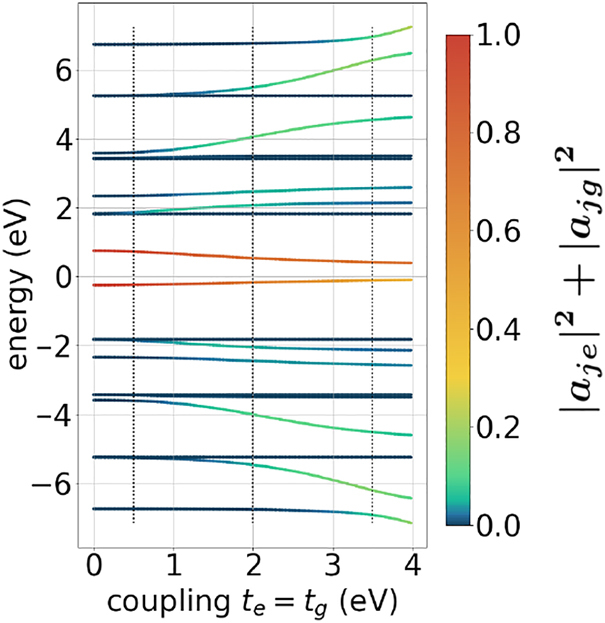
Energy levels of a hybrid system consisting of an armchair triangular graphene flake with 18 atoms and an adatom with energy levels *E*_e_ = 0.75 eV and *E*_g_ = −0.25 eV (breaking the electron–hole symmetry), located over the left top-most site of the flake. The line color represents the occupation of the adatom sites as in Figure 2.

## Appendix A. Relation of model parameters and adatom distance

For calculations in the main text, we do not specify the adatom type exactly but rather study the scaling of effects with the hopping parameters between its energy levels and the graphene flake. Here, we provide an estimate of the distances between the adatom and the graphene nanoflake that correspond to given hopping rates.

When a particular carbon atom in graphene is displaced by some distance, the hopping value is modified. We model the hopping rate *t*′ between the shifted atom and its nearest neighbor by employing a quadratic relation of the form [68]:

(13)
$$t^{\prime }=t\cdot {\left(a_{cc}/d\right)}^{2},$$

where *a*_cc_ is the distance to the nearest carbon atom in pristine graphene, and *d* is the distance from the shifted atom to the nearest carbon atom in the modified lattice. Another approximation for the hopping value modification which can be found in literature is given by an exponential relation.

We assume that the hopping rates between the graphene and the adatom levels follow a quadratic relation, analogous to Eq. (13). This allows us to estimate the distance between the adatom and flake based on the hopping value: $d=\beta a_{\alpha }\sqrt{\frac{t}{t_{\alpha }}}$ , where *a*_*α*_ is the distance at which the hopping parameter *t*_*α*_ = *t* and *β* is a parameters which scales the equilibrium distance and can account for the choice of a different adatom type than carbon. For carbon in graphene, we take *β* = 1. The left panel in Figure 13 shows the relation of the distance *d* to the hopping parameter for an adatom with carbon-like orbitals, such that *β* = 1 and *a*_*α*_ = *a*_cc_.

## B. Self-consistency procedure

To construct a ground-state density matrix of an *N*_e_-electron system, we add the density matrices corresponding to the eigenstates with energies below or equal to the Fermi energy, according to the Aufbau principle (Eq. (14)) following the Fermi–Dirac distribution and normalize its trace. In a situation without doping or degenerate eigenstates, this means creating a statistical mixture of density matrices of *N*_e_/2 lowest-energy eigenstates. The division by 2 is the consequence of the Pauli principle, allowing two electrons per state. For a bare nanoflake without doping or adatoms, the number of electrons is equal to the number of sites *N*, and the resulting zero-temperature ground state describes a uniform charge distribution across the flake, i.e., $\rho_{ll}=\frac{1}{N}$ (note that the off-diagonal elements are in general nonzero in the site basis). If we add doping electrons or attach an adatom near the nanoflake, the ground state charge distribution, which follows from the eigenstates of the tight-binding Hamiltonian, is no longer uniform. In this case, we expect some Coulomb repulsion in the initially prepared state, pushing the electrons towards a uniform distribution, which should be accounted for in the Hamiltonian. The self-consistency procedure, which allows to find the eigenstates of the system, their energies and the equilibrium charge distribution of a flake with adatom or external doping, consists of the following steps:

1. Diagonalize the Hamiltonian *H* ^(*n*)^ obtained in the previous iteration to obtain a set of energies $E_{j}^{\left(n\right)}$ and energy eigenstates |*j* ^(*n*)^⟩. In the first iteration, use *H* ^(1)^ = *H*_TB_ given in Eq. (1).
2. Calculate the density matrix *ρ* ^(*n*)^ according to the Aufbau principle:
  (14)
  $$\rho^{\left(n\right)}=\frac{2}{N_{e}}\underset{j}{\sum }f_{j}\left(N_{e}\right)|j^{\left(n\right)}〉〈j^{\left(n\right)}|,$$
  where $f_{j}\left(N_{e}\right)\in \left[0,1\right]$ is the Fermi–Dirac distribution, which determines how many electrons per spin occupy the state *j*. The thus defined density matrix is normalized as Tr *ρ* = 1.
3. For all sites, calculate the potential induced by the nonuniformity of charge distribution on the *L*th site:
  (15)
  $$\begin{array}{ll} {\left(\varphi_{\text{charge}}^{\left(n\right)}\right)}_{LL^{\prime }} & =-\delta_{LL^{\prime }}eN_{e}\underset{K}{\sum }v_{LK}\left(\rho_{KK}^{\left(n\right)}-\rho_{KK}^{0}\right). \end{array}$$
  Here, capital indices *K*, *L*, and *L*′ span over carbon sites *l* and adatom orbitals *α*. The symbol *v*_*LK*_ stands for the Coulomb interaction matrix element between sites *L* and *K*, given in Appendix D. Note that the charge-induced potential is defined similarly to the field-induced potential *φ* ^ind^ in Eq. (5): here, the charge distribution is varied with respect to the reference density matrix
  $$\rho_{ll}^{0}=\frac{1}{N_{e}}\frac{N_{e}-1}{N},\;\rho_{gg}^{0}=\frac{1}{N_{e}},\;\rho_{ee}^{0}=0,$$
  that corresponds to a uniform distribution of *N*_e_ − 1 electrons on a flake of size *N*, and one electron in the adatom’s ground state. In Eq. (5), the time-dependent charge distribution is varied with respect to the self-consistent, equilibrium charge distribution *eρ* ^sc^ obtained as the result of the procedure described here.
4. Construct a Hamiltonian including the induced charge $H^{\left(n+1\right)}=H_{TB}-e\varphi_{\text{charge}}^{\left(n\right)}$ . In practice, to ensure the convergence of the procedure, at each iteration we only include a fraction of the new induced potential and combine it with the induced potential from the previous iteration: $H^{\left(n+1\right)}=H_{TB}\;-e\left(1-p_{\text{mix}}\right)\;\varphi_{\text{charge}}^{\left(n-1\right)}\;-ep_{\text{mix}}\;\varphi_{\text{charge}}^{\left(n\right)}$ , where *p*_mix_ ∈ [0, 1]. Usually a good choice for *p*_mix_ is in the range 0.1–0.5.

Steps 1–4 are repeated with the new Hamiltonian from step 4 until self-consistency is reached, i.e., the evaluated charge-nonuniformity-induced potential *φ*_charge_ and the equilibrium density matrix *ρ* are stable. In the final step, the self-consistent Hamiltonian $H^{\text{sc}}=H^{\left(n_{\text{final}}\right)}$ is diagonalized for self-consistent energies $E_{j}^{\text{sc}}$ , eigenstate basis {|*j* ^sc^⟩}, and a self-consistent density matrix of the coupled system *ρ* ^sc^ is constructed according to the Aufbau principle in Eq. (14).

The self-consistent density matrices *ρ* ^sc^ corresponding to the charge distributions *N*_e_ *eρ* ^sc^ for adatoms attached to triangular graphene flakes 0.7 and 2.4 nm are shown in Figure 3 of the main text. Figure 4 presents the self-consistent density matrix obtained for a doped flake.

## C. Breaking the electron–hole symmetry

Figure 14 demonstrates the energy spectra of an 18-atom (0.71 nm side length) armchair-edged triangular graphene nanoflake coupled to an adatom with energy levels *E*_e_ = 0.75 eV, *E*_g_ = −0.25 eV, nonsymmetrically distributed with respect to the flake’s Fermi energy. In consequence, the electron–hole symmetry of the system is broken, leading to the splitting of resonances in the absorption spectra and more complicated dynamics with possible beating effects if the illumination frequency matches several transitions.

## D. Coulomb interaction elements

The values for on-site, nearest-neighbor and next-to-nearest-neighbor Coulomb interaction elements in a pristine graphene flake are taken from the analytical calculations performed by Potasz et al. [59] and correspond to *v*_*os*_ = 16.52 eV, *v*_*nn*_ = 8.64 eV, and *v*_*nnn*_ = 5.33 eV, respectively. For atoms which are further apart we employ the 1/*r* power law:

(16)
$$v_{ll^{\prime }}=\left\{\begin{array}{cc} v_{os} & \text{if}\;l=l^{\prime }\\ v_{nn} & \text{if}\;l\text{, }l^{\prime }\text{ are nearest neighbors}\\ v_{\text{nnn}} & \text{if}\;l\text{, }l^{\prime }\text{ are next}-\text{to}-\text{nearest neighbors}\\ \frac{E_{h}a_{0}}{\left|r_{l}-r_{l}^{\prime }\right|} & \text{otherwise } \end{array}\right.$$

where *E*_*h*_ = 27.21 eV is the Hartree energy, and *a*_0_ = 0.53 Å is the Bohr radius.

In a hybrid system consisting of a graphene flake with a coupled adatom, we use an extended Coulomb interaction matrix *v* ^hyb^. The elements on the flake part are the same as for a solo flake:

(17)
$$v_{ll^{\prime }}^{\text{hyb}}=v_{ll^{\prime }}$$

for *l*, *l*′ in the flake.

On the adatom part, we employ the on-site value:

(18)
$$v_{gg}^{\text{hyb}}=v_{ee}^{\text{hyb}}=v_{ge}^{\text{hyb}}=v_{eg}^{\text{hyb}}=v_{os}.$$

The values of elements that connect the two subsystems rely on relation (13) presented in Appendix A. Here, we assume that *β* = 1. For the coupling site *l*_*c*_ we set:

(19)
$$v_{l_{c}\alpha }^{\text{hyb}}=v_{nn}\frac{a_{cc}}{d}\sqrt{\left|\frac{t_{\alpha }}{t}\right|},$$

whereas for the other flake sites *l* (≠ *l*_*c*_):

(20)
$$v_{l\alpha }^{\text{hyb}}=v_{ll_{c}}\frac{a_{cc}}{d+a_{cc}}\sqrt{\left|\frac{t_{\alpha }}{t}\right|}.$$

Note that the proposed scaling guarantees that the Coulomb interaction between an adatom and a graphene flake will disappear as the flake is moved to infinity (when *t*_*α*_ → 0). The resulting relation of the coupling constant *t*_*α*_ with Coulomb interaction is shown in the right panel of Figure 13.

## E. Calculation of absorption spectra

The linear absorption spectrum

(21)
$$\sigma_{\text{abs}}\left(\omega \right)=\frac{\omega }{\varepsilon_{0}c}\underset{ii^{\prime }}{\sum }Im\;\alpha_{ii^{\prime }}\left(\omega \right)$$

of the system is calculated based on the method introduced in Refs [2, 3], using the dipole polarizability in frequency domain, *α*_*ii*′_(*ω*) = *p*_*i*′_(*ω*)/*E*_*i*_(*ω*). Here, *p*_*i*′_(*ω*) is the spatial component *i*′ ∈ {*x*, *y*, *z*} of the induced dipole moment that is evoked by illuminating the system with the electric field Fourier component *E*_*i*_(*ω*). In the frequency domain, the induced dipole moment reads

(22)
$$p_{i}\left(\omega \right)=\underset{L}{\sum }r_{L,i}q_{LL}^{\text{ind}}\left(\omega \right),$$

where *r*_*L*,*i*_ is the spatial component *i* of the *L*th site position and *q* ^ind^ = −*eN*_e_ *ρ* ^ind^ is the induced charge density. To obtain the Fourier component of the induced charge at site *L*, we calculate

(23)
$$q_{LL}^{\text{ind}}\left(\omega \right)=\underset{L^{\prime }}{\sum }\chi_{LL^{\prime }}\left(\omega \right)\varphi_{L^{\prime }L^{\prime }}\left(\omega \right).$$

Here, *χ* is the noninteracting susceptibility in the random phase approximation,

(24)
$$\chi_{LL^{\prime }}\left(\omega \right)=2e^{2}\underset{jj^{\prime }}{\sum }\left(f_{j^{\prime }}-f_{j}\right)\frac{a_{jL}a_{jL^{\prime }}^{⋆}a_{j^{\prime }L}^{⋆}a_{j^{\prime }L^{\prime }}}{\hbar \omega -\left(E_{j}-E_{j^{\prime }}\right)+i\hbar /2\tau }.$$

The occupations *f*_*j*_ are set to 1 for states below the Fermi energy and to 0 for states above, which is the Fermi–Dirac distribution for zero temperature. The *a*_*jL*_ coefficients are defined in Eq. (2). On the other hand, from the induced charge density, the total potential at site *L*′ is obtained from the sum of the external and the induced potential,

(25)
$$\varphi_{LL}\left(\omega \right)=\varphi_{LL}^{\text{ext}}\left(\omega \right)+\underset{L^{\prime }}{\sum }v_{LL^{\prime }}^{\text{hyb}}q_{L^{\prime }L^{\prime }}^{\text{ind}}\left(\omega \right).$$

Here, we used the Coulomb interaction matrix of the hybrid system *v* ^hyb^ that is discussed in Appendix D. The set of Eqs. (23) and (25) can be solved iteratively to arrive at the self-consistent induced charge density

(26)
$$q^{\text{ind}}\left(\omega \right)=\chi \left(\omega \right){\left(1-v^{\text{hyb}}\chi \left(\omega \right)\right)}^{-1}\varphi^{\text{ext}}\left(\omega \right),$$

whose elements are then plugged into Eq. (22) to obtain the dipole moment and, consequently, the absorption spectrum in Eq. (21).
